# Lipid Nanoparticles as Carriers for RNAi against Viral Infections: Current Status and Future Perspectives

**DOI:** 10.1155/2014/161794

**Published:** 2014-08-12

**Authors:** Josune Torrecilla, Alicia Rodríguez-Gascón, María Ángeles Solinís, Ana del Pozo-Rodríguez

**Affiliations:** Pharmacokinetic, Nanotechnology and Gene Therapy Group (PharmaNanoGene), Faculty of Pharmacy, Centro de Investigación Lascaray Ikergunea, University of the Basque Country UPV/EHU, Paseo de la Universidad 7, 01006 Vitoria-Gasteiz, Spain

## Abstract

The efforts made to develop RNAi-based therapies have led to productive research in the field of infections in humans, such as hepatitis C virus (HCV), hepatitis B virus (HBV), human immunodeficiency virus (HIV), human cytomegalovirus (HCMV), herpetic keratitis, human papillomavirus, or influenza virus. Naked RNAi molecules are rapidly digested by nucleases in the serum, and due to their negative surface charge, entry into the cell cytoplasm is also hampered, which makes necessary the use of delivery systems to exploit the full potential of RNAi therapeutics. Lipid nanoparticles (LNP) represent one of the most widely used delivery systems for *in vivo* application of RNAi due to their relative safety and simplicity of production, joint with the enhanced payload and protection of encapsulated RNAs. Moreover, LNP may be functionalized to reach target cells, and they may be used to combine RNAi molecules with conventional drug substances to reduce resistance or improve efficiency. This review features the current application of LNP in RNAi mediated therapy against viral infections and aims to explore possible future lines of action in this field.

## 1. Introduction

Gene therapy is a relatively recent approach in the management of human diseases and has resulted in an increasingly interest as therapeutic strategy. While traditional drug therapies involve the administration of therapeutic chemicals that have been synthesized outside the body, gene therapy tries to direct patient's cells to produce and deliver a therapeutic agent or to knock down the production of undesirable molecules. Gene therapy was first defined as the administration of genetic material into a human patient with the intent of correcting a specific genetic defect [1]. This definition appeared in the first gene therapy protocols in the early 90s, related to trials that aimed to correct the effects of some monogenic recessive diseases. Nowadays, the European Agency of Medicines (EMA) defines gene therapy as biological medicinal products which fulfils the following two characteristics: (a) the active substance contains or consists of a recombinant nucleic acid applied to human beings in order to regulate, repair, replace, add, or delete a genetic sequence; (b) its beneficial effect relates directly to the recombinant nucleic acid sequence it contains or to the result of genetic expression of this sequence [2].

Since the first FDA-approved gene therapy experiment in 1990 [3, 4], more than 1900 clinical trials have been performed using a number of techniques for gene therapy up to January 2014 [5].  Table 1 collects the indications addressed by gene therapy clinical trials and the number of events related to each indication. As observed in this table, infectious diseases are in the third place in the ranking, with a percentage with respect to total approved clinical trials similar to that of monogenic diseases or cardiovascular diseases.

Nucleic acid-based therapy has been traditionally focused on the use of DNA as the active substance, but since the discovery of the RNA interference (RNAi) pathway [6], RNA has also gained great interest. Among the multiple possible applications of the RNAi-based therapy, numerous studies have demonstrated its potential in the control and treatment of different viral infections in humans, such as hepatitis C virus (HCV), hepatitis B virus (HBV), human immunodeficiency virus (HIV), human cytomegalovirus (HCMV), herpetic keratitis, human papillomavirus, or influenza virus [7–13].

A key challenge to exploit the full potential of RNAi therapeutics is their efficient delivery to the target cells. On the one hand, naked RNAi molecules are rapidly digested by nucleases in the serum after systemic administration; on the other hand, due to the negative surface charge of RNA, the entry into the cell cytoplasm is hampered. A number of techniques have been attempted to overcome these problems: physical methods such as electroporation or hydrodynamic injection [14, 15], viral vectors [16], or lipid and polymeric nanoparticles [17, 18]. Among them, lipid nanoparticles (LNP) represent one of the most widely studied delivery systems for* in vivo* application of RNAi [19, 20]. The aim of this review is to collect the state of the art and the future perspectives of the utility LNP as RNAi vectors for human viral infections.

## 2. RNA Interference (RNAi)

**Figure 1:**
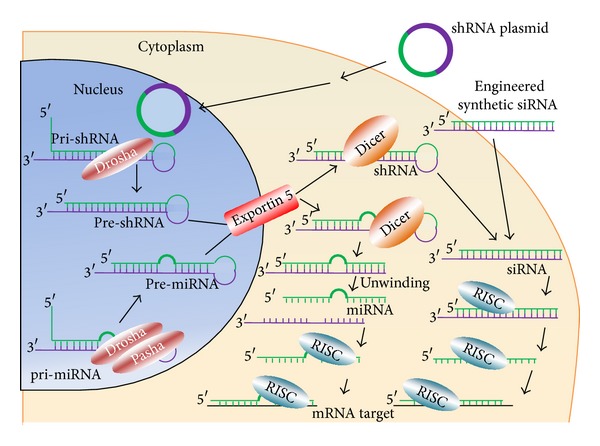
RNAi mechanism. Differences between siRNA, shRNA, and miRNA as therapeutic tools.

RNAi is a naturally occurring process of gene regulation present in plants and mammalian cells. The first evidence of the existence of this mechanism appeared in 1998, when Fire et al. [6] observed in* Caenorhabditis elegans *that double-stranded RNAs (dsRNAs) were the basis of sequence-specific inhibition of protein expression. Subsequent works demonstrated that the molecules that induced RNAi were short dsRNAs, of 21 nucleotides in length, called short interfering RNAs (siRNAs), and that siRNAs were able to start the RNAi process in mammalian cells [21]. The RNAi response is activated when the dsRNA is processed by a ribonuclease III-like enzyme called Dicer, resulting in the formation of a siRNA. The siRNA is incorporated into the RNA induced silencing complex (RISC), where a helicase unwinds the duplex siRNA. The resulting antisense strand guides the RISC to its complementary mRNA, which will be cleaved [22]. Typically, there are three different types of commonly used RNAi molecules: siRNA, short-hairpin RNA (shRNA, also named expressed RNAi activators), or microRNA (miRNA). siRNAs, as mentioned before, are dsRNA molecules of about 19–23 base pair nucleotides in length, able to mediate site-specific cleavage and destruction of the targeted mRNA [23]. shRNAs consist of two complementary 19–22 bp RNA sequences linked by a short loop of 4–11. These RNAs are synthesized within the cell by DNA vector-mediated production [24]. shRNAs can be transcribed through either RNA polymerase II or III. The first transcript generates a hairpin like stem-loop structure and then is processed in the nucleus by a complex containing the RNase II enzyme Drosha. The individual pre-shRNAs generated are finally transported to the cytoplasm by exportin 5. Once in the cytoplasm, the complex Dicer processes the loop of the hairpin to form a double-stranded siRNA. shRNAs represent an important tool in the assessment of gene function in mammals and are largely used as a research tool. miRNAs are single stranded RNAs of about 20–24 nucleotides. This kind of RNAs acts as endogenous posttranscriptional repressors to downregulate gene expression [25]. miRNAs are transcribed from DNA as primary miRNA (pri-miRNA); in this case, pri-miRNAs are processed into precursor miRNA (pre-miRNA) by two proteins: Drosha and Pasha. Pre-miRNAs are then transported to the cytoplasm, and after processing by Dicer and unwinding to obtain the miRNA, the following steps are identical to those that occur with siRNA and shRNA as it is illustrated in Figure 1. Recent observations have shown that both miRNAs and siRNAs can suppress translation of mRNAs (in the case of an imperfect match) and can cleave target RNAs (in the case of a perfect match) and play a decisive role in gene and genome regulation [26]. It has been also observed that shRNA can act via miRNA- or siRNA-like mechanism. It is believed that the miRNA-like mechanism is faster than siRNA-like mechanism, because the latter acts via perfect complementarity for a target message [27].

The main advantage of synthesized siRNAs is that these molecules do not need to reach the nucleus to exert effect. However, modifications are necessary to increase their stability, resulting in some cases in loss of siRNA function [28]; there must be a balance between stability and efficacy when modifications are introduced in siRNA. When miRNAs or shRNA expression plasmids are used, they must initially reach the nucleus of cells to be processed. The resulting molecules are then transported to the cytoplasm and finally incorporated into the RISC for activity. Therefore, a limitation of miRNA and shRNA is the need to be delivered into the cell nucleus, although they have a greater durability and higher silencing capacity than siRNAs [29].

RNAi seems to play an important role in the antiviral defense mechanism in human cells, suggesting its potential use as therapeutic in human infectious diseases. For instance, HIV-1 shows higher replication capacity in cells that have suffered knockout of Dicer and Drosha expression [30]. In this sense, the mammalian stomatitis virus achieved increased accumulation in* Caenorhabditis elegans* with defective RNAi machinery [31]. It has been also observed that the interferon (IFN) pathway works in coordination with miRNA to control viral infections. IFN-*β* can induce the expression of several cellular miRNAs that form almost perfect nucleotide base pair matches with the HCV genome. When these miRNAs are artificially introduced, the antiviral effects of IFN-*β* in HCV are reproduced, and the IFN response is lost when they are experimentally removed [32].

As mentioned above, to take advantage of the potential of RNAi as antiviral therapy, effective delivery is essential; in this regard, lipid-based systems are being widely used as RNAi-delivery vectors.

## 3. Lipid Nanoparticles (LNP)

Lipid-based systems have been increasingly recognized as one of the most promising delivery systems for RNAi. Lipid carriers may be available in solid, semisolid, or liquid state in the form of solid lipid nanoparticles, nanostructured lipid carriers, lipid drug conjugate nanoparticles, liposomes, or nanoemulsions. These lipid-based systems were initially designed to address some of the challenges of conventional drug delivery systems, such as the increase of bioavailability of poorly soluble drugs, among others. Nowadays, the application of LNP in other fields, such as gene therapy, has gained attraction.

RNAi-lipid-based nanocarriers are able to provide protection from serum nucleases and extended circulation, which results in a higher access to the target tissue [38]. Sometimes, targeting is achieved by surface modification of nanocarriers with specific ligands to target cell populations, such as mannan-modified nanoparticles to direct vectors to alveolar macrophages [39]. Once in the target tissue, the RNA-delivery system will be internalized by the target cell and, upon receptor-mediated endocytosis, will be able to escape from the endosomal compartment into the cell cytoplasm where RNA machinery is located, while avoiding lysosomal enzymes [40]. When these delivery systems are applied to the treatment of viral infections, multiadministration treatment modalities are possible for improved clinical outcomes [41]. Moreover, due to their biocompatibility and their ease of large-scale production, large batches with reproducible specifications are possible.

### 3.1. Solid Lipid Nanoparticles (SLNs)

SLNs are considered to be one of the most effective lipid-based colloidal carriers. SLNs are in the submicron size range of 50–1000 nm and are composed of physiologically compatible lipids recognized as safe, which are in solid state at room temperature. They consist of a solid lipid core surrounded by a layer of surfactants in an aqueous dispersion, with multiple potential combinations of lipids and surfactants [42, 43]. The interest on SLNs has led to the development of different types of production methods (i.e., high-pressure homogenization) successfully implemented in pharmaceutical industry. The SLNs obtained with these techniques show long-term stability and the possibility to be subjected to commercial sterilization and lyophilized procedures [44–46].

SLNs are used not only as conventional drug delivery systems but also as carriers for therapeutic peptides, proteins or antigens, and bioactive molecules. Mannosylated SLNs loaded with hepatitis B surface antigen (HBsAg) were subcutaneously administered* in vivo* in mice and sustained antibody titer was obtained; these results demonstrated the potential of SLNs as carriers for vaccine delivery against HBV [59].

As gene delivery systems, SLNs have been studied over the last years for a large number of diseases [46, 60, 61] and different routes of administration [62]. Cationic lipids are used to prepare SLNs due to their positive surface charge that interacts electrostatically with the negative charge of the nucleic acids. Our research group has developed nonviral vectors based on cationic SLNs [63, 64] decorated with peptides [65, 66], dextran [60], oligochitosans [67], or hyaluronic acid [68] able to transfect several cell lines and tissues both* in vitro* and* in vivo* [69–71]. To date, vectors that were developed for DNA delivery are being applied for siRNA delivery. In spite of their different physicochemical properties and the need to be transported to different parts of the cell (plasmid DNA needs to be transported into nucleus for gene expression whereas siRNA reaches its target in the cytoplasm), a number of publications describe the use of SLNs for delivery of both DNA [39, 72–74] and RNAi mediated molecules [75–78] with successful results.

Focusing on RNAi delivery, Montana et al. [78] demonstrated the utility of cationic SLNs, produced by microemulsion using Compritol ATO 888 as matrix lipid, Pluronic F68 as tensioactive, and dimethyldioctadecylammonium bromide (DDAB) as cationic lipid, as RNA carriers. In another study [76], SLNs composed of cholesteryl ester, triglyceride, cholesterol, dioleoylphosphatidylethanolamine (DOPE), and 3beta-[N-(N′,N′-dimethylaminoethane) carbamoyl]-cholesterol (DC-chol) were able to bind electrostatically siRNA conjugated with polyethylene glycol (PEG) (siRNA-PEG). When compared to polyethylenimine, a commonly used transfectant polymer, the SLN-based system showed similar gene silencing efficiency and very low cytotoxicity in PC3 (human prostate cancer cell line) and MDAMB435 (human breast cancer cell line) cells. In a later work, these SLNs containing a siRNA anti-c-Met (an oncogene overexpressed in a variety of carcinomas) were evaluated* in vivo* in a glioblastoma multiform mouse model. After intravenous administration to mice, the siRNA-PEG/SLN system specifically crossed the blood-brain barrier to the tumor site with no apparent systemic toxicity. Treatment significantly inhibited tumor growth in a dose dependent manner, and a downregulation of c-Met was observed [77].

### 3.2. Nanostructured Lipid Carriers (NLCs)

NLCs are solid lipid core carriers in the nanometer range composed of a mixture of liquid and solid lipids that are spatially incompatible leading to special structures with improved drug encapsulation and release properties. NLCs were developed as a tool to overcome the drawbacks of SLNs and to increase the oral bioavailability of poorly soluble compounds [79–83].

The choice of the lipid is critical to ensure the stability of the drug [79], and the structure of the lipid core matrix determines the classification of NLCs. Imperfect NLCs are made by mixing small amounts of liquid lipid and fatty acids with different chain lengths as solid lipid. The imperfections that are generated in the lipid core due to the crystallization increase the drug load and reduce, although not completely, the drug expulsion [84, 85]. Multiple NLCs are made by mixing solid lipids and an excess of liquid lipid, generating oily nanocompartments into the lipid matrix where the drug is well accommodated [44]. Finally, in structureless or also called amorphous NLCs, special liquids are used, and the expulsion of the drug is avoided due to the lack of crystallization [85].

The application of NLCs to the treatment and prevention of infectious diseases has been attempted in various studies focused on the development of new antimalarial treatments [86–88] or in the improvement of encapsulation of hydrophilic antiretroviral drugs [89–91]. Although there are few reports about the application of NLCs as RNAi delivery systems, they could be an interesting alternative in codelivery strategies with conventional drugs. For instance, Taratula et al. [92] have recently described a multifunctional NLC system to improve the efficacy of anticancer drugs in lungs. The system was composed of an anticancer drug (doxorubicin or paclitaxel), joint with siRNA to avoid cellular resistance (siRNA targeted to MRP1 mRNA as a suppressor of pump drug resistance and siRNA targeted to BCL2 mRNA as a suppressor of nonpump cellular resistance) and an analog of luteinizing hormone-releasing hormone as a targeting moiety to lungs. The inhalation of the NLCs by an orthotopic mice model of human lung cancer resulted in efficient suppression of tumor growth and prevention of adverse side effects on healthy organs.

### 3.3. Lipid Drug Conjugates (LDCs)

LDCs are the most accepted lipid-based nanoparticle systems for the delivery of hydrophilic drugs, as they improve the drug loading capacity of SLNs and NLCs. In LDC nanoparticles, hydrophilic drugs are first conjugated with lipid components by covalent linking between an amino group or a hydroxyl group of the drug and carboxyl groups of the lipid core to obtain a lipophilic complex [93]. The water insoluble LDC is converted to nanoparticles by means of traditional methods used to prepare SLNs or NLCs [94, 95].

LDCs have shown hopeful results as delivery system for hydrophilic antitrypanosomal drugs [96, 97], for oral application of methotrexate [94], and, more recently, for chemotherapy agents [1, 95], such as decitabine, a drug that shows low oral bioavailability [98]. Neupane et al. [99] developed LDC nanoparticles of decitabine to increase its permeability and protect against chemical degradation. LDCs were obtained by salt formation of DCB with stearic acid to be formulated as LDC nanoparticles by cold high-pressure homogenization after addition of surfactants Tween 80, Poloxamer 188, and Labrasol.* Ex vivo* gut permeation studies proved that the drug in LDC nanoparticles showed nearly fourfold increase in the apparent permeability coefficients with respect to the plain decitabine.

### 3.4. Cationic Emulsions

Emulsions are dispersions of one immiscible liquid in another stabilized by a third component, the emulsifying agent [100]; therefore, they present in their composition three components: oil, water, and surfactants. When cationic surfactants are used, these dispersed systems make them suitable for gene delivery. The presence of cationic surfactants causes the formation of positively charged droplets that promote strong electrostatic interactions between emulsion and the anionic nucleic acid phosphate groups. Cationic emulsions composed of cationic lipids and core oil have been shown to be useful for gene delivery [101, 102]. The colipid DOPE is largely used to improve the ability of cationic emulsions and liposomes to transfect cells due to its fusogenic properties. This can be partially explained by the fact that the amine group of DOPE interacts with DNA phosphate groups, thus weakening the binding affinity between cationic lipids and DNA [100].

In spite of the advantages of nanoemulsions for delivery of nucleic acids, only few attempts have been made to use this new delivery system for RNAi. For instance, Kaneda et al. [103] showed the potential application of cationic nanoemulsion prepared with DOTAP, DOPE, and cholesterol for siRNA delivery. Transfection complexes, with a mean particle diameter of approximately 300 nm, were able to suppress endothelial cell expression of upregulated vascular adhesion molecules. To the knowledge of the authors, no studies about cationic emulsions with RNAi molecules to treat viral infections have been published.

### 3.5. Liposomes

Liposomes are colloidal lipid- and surfactant-based delivery systems, composed of a phospholipid bilayer surrounding an aqueous compartment. They may present as spherical vesicles and can range in size from 20 nm to a few microns. Cationic lipid-based liposomes are able to complex with negatively charged nucleic acid via electrostatic interactions, resulting in complexes that offer biocompatibility, low toxicity, and the possibility of large-scale production required for* in vivo* clinical applications [104]. The lipid to RNA ratio and overall lipid concentration used in forming these complexes are very important for efficient gene delivery and vary with applications. Liposomes can fuse with the plasma membrane for uptake; once inside the cell, the liposomes are processed via the endocytic pathway and the genetic material is then released from the endosome/carrier into the cytoplasm. Compared to polymeric nanoparticles, liposomes have long been perceived as better drug delivery vehicles because of their superior biocompatibility, as liposomes are basically analogues of biological membranes, which can be prepared from both natural and synthetic phospholipids [104].

Neutral lipids are highly nontoxic and do not activate an immune response [105]. 1,2-Oleoyl-sn-glycero-3-phosphocholine (DOPC) and DOPE are among the most widely used neutral lipids. Simply mixing siRNA with DOPC results in high encapsulation efficiency [106]. However, neutral liposomes yield relatively low transfection efficiency. Cationic lipids, such as 1,2-dioleoyl-3-trimethylammonium-propane (DOTAP), can complex electrostatically with siRNAs and be used to create a more effective liposome as the positively charged lipids provide enhanced cell entry and increased protection against serum enzymes [107].

Although liposomes are one of the most commonly used transfection reagents* in vitro*, safe and efficacious delivery* in vivo* is more difficult to achieve due to toxicity, nonspecific uptake, and unwanted immune response. Much of the nonspecific response and toxicity is directly linked to the positive charge on the surface of the particles necessary for the binding of oligonucleotides. In order to improve their behavior, in recent years, significant effort has been dedicated to modifying the composition and chemical structure of liposomes. For instance, different additives, such as hydrophobic cholesterol, nonionic surfactants, or PEG, can be used to enhance* in vivo* stability after exposure to blood components [108]. It has been reported that the stability-enhanced liposomes have much better transfection efficiencies, especially under* in vivo* conditions [109]. To increase specificity, PEGylated immunoliposomes conjugated with targeting ligands have been developed [110].

Liposomes have been widely studied as RNAi carriers as potential treatment of viral infections such as HCV, HBV, and VIH, among others [33, 34, 48, 49, 111].

## 4. Lipid Nanoparticles as RNAi Carriers against Viral Infections

As mentioned above, an effective delivery system will have to be developed for exploiting the gene silencing by RNA interference strategy for antiviral therapy. The applicability of LNP for the treatment of viral infections is emerging in the last years. On the one hand, the use of LNP as nonviral nanocarriers of gene material is the most studied approach. In this sense, some researchers suggest the simultaneous administration of different siRNAs or shRNAs to avoid the important payload of viral mutation escape [112]. On the other hand, the prophylaxis for viral infections using RNAi has been considered a promising strategy.

The RNAi process requires a high specificity of gene sequences and the choice of the target is the most crucial step for a successful viral inhibition. It is very important to design siRNA against very highly conserved sequences of the viral genome in order to optimize efficacy in inhibiting a majority of virus straints. In addition to its utility as a stand-alone strategy, RNAi may have expanded applications as an adjuvant in multipronged treatment settings. Another RNAi adjuvant strategy is the use of dsRNA oligonucleotides as immunostimulatory agonists alongside vaccines, as in the case of a RIG1 agonist to enhance the activity of a DNA vaccine against influenza [113].

### 4.1. Hepatitis C Virus (HCV)

According to the World Health Organization (WHO), every year, 3-4 million people are infected with the HCV, and about 150 million people are chronically infected, which can lead to liver cirrhosis and/or hepatocellular carcinoma (liver cancer). In consequence, more than 350000 people die from hepatitis C-related liver diseases every year [114].

Combination of generic antiviral agents, IFN-*α* and ribavirin, is the mainstay of current hepatitis C treatment. Unfortunately, IFN-*α* is not available in some countries, people do not always tolerate well this drug, and many people do not finish their treatment. In addition, some virus genotypes do not respond well to IFN-*α* [115]. Recently new antiviral drugs, telaprevir or boceprevir, have been added to set up the so-called triple therapy; the experience in patients with chronic hepatitis C genotype 1 shows that this new combination is superior to dual therapy in terms of sustained virologic response [116]. However, genotype independent alternatives should be more effective in the treatment and prevention of this liver infection. In this regard, the RNAi technology is an attractive strategy.

HCV is a RNA virus belonging to Flaviviridae family. The single-stranded RNA genome of this virus acts also as mRNA, which makes it an attractive target for RNAi-based therapy. Several works show potent HCV replication inhibition by using chemically synthesized siRNAs targeted against sequences in the protein-coding regions of core, E2, NS3, NS5B, or NS4 [117–119]. However, these viral coding sequences suffer variations among different HCV genotypes. Therefore, highly conserved regions, such as 5′ untranslated regions (5′UTR), seem to be better targets for developing a rational antiviral strategy [120]. The replication of different HCV genotypes has been inhibited by targeting 5′UTR with synthetic siRNA or shRNA expression [121–123]. The main limitation of all these new strategies lies in the emergence of resistant virus variants due to the high specificity of RNAi and the prolonged treatment. In order to prevent escape mutants, some approaches have been proposed. The internal ribosome entry site (IRES) in the 5′UTR is required for the synthesis of viral proteins and, therefore, for viral replication. Mutations in these structures might lead to loss of function [9], making IRES an excellent target for anti-HCV drugs, which might prevent viral escape; it has also been targeted by the RNAi technology [124]. Another possibility to reduce resistant variants is the combination of two or more RNAi molecules with different specificities [125] targeted to separated regions of the HCV genome. Targeting multiple sites of the HCV genome and host genes necessary for virus replication has also been documented as a valid approach to prevent the development of resistance [126, 127].

Among the RNAi delivery systems against HCV, cationic lipid-based nanoparticles play an interesting role; these lipid-based vectors are well characterized as nanocarriers for systemic delivery of RNAi molecules to the liver, due to their safety profile and simplicity of production.  Table 2 summarizes the strategies employed by different authors to improve the efficacy of lipid nanosystems in RNAi-mediated therapies against HCV.

Watanabe et al. [33] designed lactosylated cationic liposomes to deliver siRNA targeting the 5′-UTR and 3′-UTR of the HCV genome into mouse liver hepatocytes; the galactose terminus of lactose is a ligand of the asialoglycoprotein receptor, which is specifically expressed in the surface of hepatocytes. Intravenous administration of the mentioned vectors into HCV-transgenic mice resulted in a decrease of HCV core protein.

Another possibility is the functionalization of LNP with apolipoproteins. Kim et al. [34] developed liver-specific siRNA delivery vectors composed of cationic liposomes and apolipoprotein A-1 (apo A-1) derived from human plasma. This protein, component of high density lipoprotein (HDL), has been proposed as a targeting ligand to hepatocytes [48]. After intravenous administration of the lipid-based systems containing HCV-core specific siRNA into an HCV mouse model, viral expression was inhibited by 65–75% in the liver on day 2. In the same work, the chemical modification of the HCV-core specific siRNA to increase its serum stability resulted in gene silencing efficacy up to 95% for at least 6 days. In order to avoid safety problems related to the risk of pathogen contamination associated with the use of plasma-derived protein, in a posterior work [35] apo A-1 was substituted by a recombinant human apo A-1 (rhapo A-1). This new apolipoprotein of low endotoxin grade was expressed and purified from an* Escherichia coli* expression system. The use of rhapo A-1 to deliver siRNA to the liver resulted as effective and selective as plasma-derived apo A-1, without affecting the normal liver function.

The choice of an adequate RNAi molecule is also crucial. HCV replication has also been inhibited both* in vitro* and* in vivo* by delivery of different siRNAs with lipid nanosomes composed of the cationic lipid DOTAP, the helper lipid cholesterol, and the peptide protamine. Nanosomes are nanosize unilamellar vesicles obtained by high-pressure homogenization of lipid dispersions [36, 128]. In a first work, nanovectors were optimized in terms of lipid-to-siRNA ratio and favorable particle size depending on sonication time. Cell viability was maintained about 90% and HCV inhibition reached approximately 85% [128]. Later on, the cationic lipid nanosomes were used to complex different siRNA targeted to the 5′UTR of the HCV genome. Chandra et al. compared repeated treatments with two-siRNA versus a single siRNA treatment. A reduction in the development of resistant mutants to the siRNA therapy was observed when the combinatorial strategy was used, and after systemic injection in a liver tumor-xenotransplant mouse model of HCV, significant inhibition of virus replication was obtained [36]. More recently, short synthetic shRNAs (sshRNAs) that target a sequence within IRES have been formulated into LNP by the process of step-wise ethanol dilution and spontaneous vesicle formation. sshRNA was dissolved in an aqueous solution containing 30% ethanol and added to preequilibrated LNP at 35°C. After reaching the final sshRNA to lipid ratio, the mixture was incubated for further 30 min at 35°C to allow vesicle reorganization and encapsulation of the RNA. Finally, LNP were dialyzed against PBS and filter sterilized through a 0.2 *μ*m filter. The intravenous injection of this vector resulted in enough uptake by the hepatocytes to substantially suppress gene expression in a rapid and durable manner [37].

### 4.2. Hepatitis B Virus (HBV)

WHO estimates that about 600,000 people die every year due to the consequences of HBV infection (mainly cirrhosis of the liver and liver cancer). A vaccine against hepatitis B has been available since 1982. Hepatitis B vaccine is 95% effective in preventing infection but offers scarce therapeutic benefit to chronic carriers [129]. Some people with chronic hepatitis B are treated with drugs, including IFN, nucleotide/nucleoside analogues, and immunomodulators, but there is no specific treatment for acute hepatitis B [130]. Moreover, current therapies have limited efficacy, are expensive, produce side effects, and are associated with viral resistance [131]. The emergence of resistance has been reduced by new antivirals (entecavir or tenofovir), but patients usually need to take these drugs for life, because interruption of treatment quickly reactivates viral replication [132]. Therefore, there is need for finding effective therapies against HBV, with the use of RNAi being an attractive possibility. RNAi has also been studied as a possible vaccine against HBV [133], but its main application is the treatment of chronic HBV infection.

HBV viron contains a partly double-stranded relaxed circular DNA (rcDNA) that is encapsidated by core proteins and enveloped with S proteins and membrane lipids from the host to form viral particles [134]. When a hepatocyte is infected endogenous repair enzymes convert rcDNA in a fully double-stranded, circular, and supercoiled DNA (cccDNA), which serves as template for transcription of HBV RNA. Expression of viral proteins and viral replication may be potentially knocked down by RNAi-based therapeutics.

RNAi activators used against HBV include expressed or engineered synthetic RNAi intermediates; each class of silencing molecules has advantages and disadvantages. Expressed anti-HBV sequences, as plasmids that produce specific siRNAs [135] or as shRNA [136–140], have demonstrated efficacy* in vitro* and* in vivo* and they achieve more sustained silencing effect, whereas synthetic siRNAs require repeated administration to provide a long-term suppression of HBV replication [141–143]. However, expressed RNAi activators are complex in terms of delivery and dose control, which have led to the development of chemically modified synthetic siRNAs with the aim of improving silencing efficacy, specificity for a particular target mRNA, avoidance of innate immunostimulation, stability, and delivery to target tissue [51, 144].

The delivery of these RNAi molecules against HBV has been addressed by several research groups by means of cationic lipid-based systems, recapitulated in Table 3. In an early study, Morrissey et al. [47] entrapped a couple of chemically modified synthetic anti-HBV siRNAs in stable nucleic-acid-lipid particles (SNALPs). SNALPs consist of a lipid bilayer composed of cationic and fusogenic lipids, which are coated with a PEG-lipid. Inner lipids enable cellular uptake and endosomal release of the molecules payload, whereas the coating stabilizes the particles during formulation and shields the particles* in vivo* to avoid rapid systemic clearance. Following administration, the PEG-lipid dissociates from the nanoparticles and the SNALPs become a transfection-competent entity [145]. After intravenous administration of SNALPs-siRNA vectors to mice carrying the replicating virus, encapsulated siRNA presented a longer half-life in plasma and liver than nonencapsulated. Sustained specific reduction in HBV titers, for up to 7 days, and reduced toxic and immunostimulatory side effects were achieved [47]. Like in the case of HCV, in order to improve liver-targeting, apo A-1 has been combined with the cationic lipid DOTAP and the helper lipid cholesterol to form lipoplexes with siRNAs against HBV [48]; apo A-1 was incorporated into the formulation by reassembling the liposomes with a solution of the protein at 4°C overnight. Intravenous injection of these lipoplexes into a HBV mouse model significantly reduced viral protein expression during at least 8 days in only a single treatment. In two subsequent studies, lipid-based systems demonstrated more effective or comparable inhibition of viral proliferation than lamivudine, a licensed HBV drug [49, 50]. In one case [49], lipoplexes were prepared by adding an aqueous solution of siRNA targeting conserved regions of the HBV genome to a dispersion of lipid vesicles with constant vortex mixing. Thereafter, a PEG-lipid was added to the lipoplexes and the mixture was incubated for 16 h. These vectors were also lyophilized in presence of trehalose for long-term storage. In the second case [50], the siRNA targeting conserved regions of the HBV genome was combined with the cationic lipid N′,N′-dioctadecyl-N-4,8-diaza-10-aminodecanoylglycine amide (DODAG) under conditions of rapid vortex mixing to produce siRNA-DODAG nanoparticles. More recently, a liver-targeting cholesterol galactoside was incorporated into lipoplexes to obtain an altriol-modified siRNA delivery system [51]. The galactose-conjugated cholesterol was synthesized by a cooper-mediated “click” reaction between the 2-propynylcarbamate derivative of cholesterol and* O*-tetraacetate galactose azide. This lipid was mixed with a nucleic acid binding cholesterol derivative and the helper lipid to obtain liposomes capable of binding the siRNA. The improved hepatotropism and attenuated immunostimulatory properties of the vectors demonstrated that galactose functionalization has also potential for delivery of RNAs to hepatocytes in the treatment of hepatic viral infections and other liver diseases.

### 4.3. Human Immunodeficiency Virus (HIV)

HIV continues to be a major global public health issue, having claimed more than 36 million lives so far. In 2012, approximately 35.3 million people lived infected by HIV [146]. Although effective treatment with antiretroviral drugs can control the virus, there is no cure for HIV infection; therefore, novel and more promising strategies must be developed.

HIV belongs to retroviruses, which are viruses that contain RNA as genetic material. Throughout its life cycle, the RNA of HIV is not protected during viral uncoating and reverse transcription. At this point, therapeutic RNAi molecules may interact with viral RNA and blockage or reduce HIV infection. Synthetic and expressed RNAi have been evaluated preclinically to target HIV-encoded RNA such as rev, nef, or integrase [147–149] or host factors, mainly the chemokine receptor 5 (CCR5) [111, 150, 151], a promising target because it is apparently not important for human physiology [152]. Some of these targets have also been evaluated in the clinical practice, by using lentiviral or* ex vivo* strategies, but the drawbacks related to immunogenicity of the vectors and/or inconveniences for patients call for new delivery systems [153–156]. In addition, as with other viruses, when only one RNAi is targeted escape mutants can be generated [157]; targeting multiple viral targets, combinations of host and viral proteins, even combining host, viral proteins, and clinically approved antiretroviral drugs have demonstrated improved efficacy against HIV [152, 158–160].

In the field of vaccines against HIV, RNAi may also play an important role. On the one hand, RNAi technology has been proposed as a strategy to block genes related to the suppression of immune response mediated by DNA vaccines, which are limited in nonhuman primates and humans, probably due to the relative brief duration of vaccine antigen expression* in vivo*. After intramuscular (i.m.) administration of plasmid DNA in mice, an adaptive immune response that mediates the apoptotic destruction of vaccine antigen-expressing myocytes was detected [161]. The use of a shRNA targeted to caspase-12, a cell death mediator activated after plasmid DNA vaccination, resulted in increased HIV-gp120 Env antigen expression and higher CD8 T cell and antibody responses [162]. On the other hand, siRNA can be used in a prophylactic manner. For instance, siRNAs designed to knock down CCR5 and/or viral genes in CD4+ T cells, macrophages, and dendritic cells have shown protection capacity against HIV vaginal transmission when applied intravaginally to humanized mice [163]. In this regard, LNP may be the delivery system of choice, as they have demonstrated adjuvant properties for HIV vaccine. Anionic SLNs prepared with an emulsifying wax and coated with the HIV-Tat (transactivator of transcription) protein and administered subcutaneously twice to mice at an interval of 2 weeks elicited IgG and IgM responses similar to the commonly used adjuvant Alum and a higher release of IFN-*γ* in splenocytes [164]. In addition, anti-Tat IgG titers obtained with Alum carrying Tat were lower than those obtained with a reduced dose of the peptide adjuvanted with the SLN [165]. Other administration routes have also been explored. Intradermal and intranasal administration in mice of carnauba wax based SLN coated with HIV gp140 antigen and toll-like receptor-9 (TLR-9) yielded higher systemic and mucosal immunity than the antigen alone [166]. In another work, liposomes bearing anti-CCR5 siRNA were functionalized with the monoclonal antibody (mAbd) against lymphocyte function-associated antigen-1 (LFA-1) integrin. After intravenous administration into humanized mice, leukocyte-specific gene silencing was obtained during 10 days, and when challenged with HIV plasma viral load and loss of CD4 T cells were reduced [111].

### 4.4. Other Viral Infections

Although HCV, HBV, and HIV are the most studied, RNAi with lipid formulations has also been applied to the potential treatment of other viral infections such as herpes simplex virus, Ebola virus, human papillomavirus (HPV), or rabies virus, among others.

Herpes simplex virus-2 (HSV-2) infection causes significant morbidity and is an important cofactor for the transmission of HIV infection. In a study carried out by Palliser et al. [167], seven siRNAs targeting three essential HSV-2 genes (UL5 (a component of the helicase-primase complex), UL27 (envelope glycoprotein B), and UL29 (a DNA-binding protein)) were prepared and assayed for viral protection. siRNAs lipoplexes were efficiently taken up by epithelial and lamina propria cells and silenced gene expression in the mouse vagina and ectocervix for at least nine days. Intravaginal application of siRNAs targeting the HSV-2 UL27 and UL29 genes was well tolerated, did not induce IFN-responsive genes or cause inflammation, and protected mice when administered before and/or after lethal HSV-2 challenge. In a later study [168], one of the viral siRNAs was combined with a siRNA targeting the HSV-2 receptor nectin-1. Cholesterol-conjugated- (chol-) siRNAs silenced gene expression in the vagina without causing inflammation or inducing IFNs. The viral siRNA prevented transmission within a day of challenge, whereas the siRNA targeting the HSV-2 receptor nectin-1 protected for a week, but protection is delayed for a few days until the receptor is downmodulated. Combining siRNAs targeting a viral and host gene protected mice from HSV-2 for a week, irrespective of the time of challenge.

Ebola virus (EBOV) infection causes a frequently fatal hemorrhagic fever that is refractory to treatment with currently available antiviral therapeutics. Geisbert et al. [169] prepared four siRNAs targeting the polymerase gene of the Zaire species of EBOV (ZEBOV) and complexed them with polyethylenimine or formulated them in SNALPs. Guinea pigs were treated with these siRNAs either before or after lethal ZEBOV challenge. Treatment of guinea pigs with a pool of the L gene-specific siRNAs delivered by polyethylenimine polyplexes reduced plasma viremia levels and partially protected the animals from death when administered shortly before the ZEBOV challenge. Evaluation of the same pool of siRNAs delivered using SNALPs proved that this system was more efficacious, as it completely protected guinea pigs against viremia and death when administered shortly after the ZEBOV challenge. Additional experiments showed that 1 of the 4 siRNAs alone could completely protect guinea pigs from a lethal ZEBOV challenge. In a later study [170], the same research group assessed the efficacy of modified nonimmunostimulatory siRNAs in a uniformly lethal nonhuman primate model of ZEBOV haemorrhagic fever. Two (66%) of three rhesus monkeys given four postexposure treatments of the pooled anti-ZEBOV siRNAs were protected from lethal ZEBOV infection, whereas all macaques given seven postexposure treatments were protected. The treatment was well tolerated with minor changes in liver enzymes that might have been related to viral infection. From results obtained in these studies, in January 2014, a Phase I clinical trial commenced, as it will be mentioned in Section 5 of this review.

The human papillomavirus (HPV) is the etiologic agent of cervical cancer. The E7 gene is a promising target for RNAi application in the management of HPV infection [171]. It plays an important role in cell-cycle regulation. Moreover, E7 inhibits the therapeutic effect of antiviral agents (such as IFN-*α*) and suppresses local immunity through functional inhibition of antigen-presenting cells and cytotoxic T lymphocytes. E7 gene silencing in high risk HPV types such as HPV-16 and HPV-18 by siRNAs suppressed cell growth* in vitro* and* in vivo*. siRNA duplexes or shRNA-expressing plasmids targeting the E7 genes of HPV-6b or HPV-11 were inoculated into cultured E7-expressing cells via cationic liposomes or into E7 gene-expressing mouse tumor models intratumorally or intravenously [170]. Both siRNAs and shRNA-expressing plasmids reduced* in vitro* the mRNA levels of HPV-6b or HPV-11 E7 to 20–40%. E7 mRNA expression in tumor models was reduced to 45–50% after three intratumoral injections. Intratumoral injections of RNAi effectors induced greater inhibition than did intravenous injections.

Rabies virus (RABV) infection continues to be a global threat to human and animal health, yet no curative therapy has been developed. siRNAs that target the conserved region of the RABV challenge virus standard (CVS)-11 strain nucleoprotein gene represent a promising approach for treating RABV infections [172]. Using a plasmid-based transient expression model, these siRNAs were capable of significantly inhibiting viral replication* in vitro* and* in vivo*. They effectively suppressed RABV expression in infected baby hamster kidney-21 (BHK-21) cells, as evidenced by direct immunofluorescence assay, viral titer measurements, real-time PCR, and Western blotting. In addition, liposome-mediated siRNA expression plasmid delivery to RABV-infected mice significantly increased survival, compared to a nonliposome-mediated delivery method.

siRNA immunoliposomes have also been shown as a therapeutic agent against H5N1 influenza virus infection. In a recent study [173], siRNA specific for influenza virus nucleoprotein (NP) mRNA was employed as the key antiviral agent to inhibit viral replication. A humanized single-chain Fv antibody (huscFv) against the hemagglutinin (HA) of H5N1 highly pathogenic avian influenza virus (HPAI) was used as the targeting molecule to HA of H5N1 virus, which is abundantly expressed on the surface of infected cells (the HA target cells). The immunoliposomes were shown to specifically bind HA-expressing Sf9 cells and demonstrated enhanced siRNA transfection efficiency. Furthermore, the siRNA silencing effect was more pronounced when the immunoliposomes were administered 6 to 12 h after H5N1 infection in MDCK cells compared with the nontargeted liposomes.

In a recent study [174], liposomes were used to deliver a self-amplifying RNA vaccine for respiratory syncytial virus (RSV). The vaccine potently induced neutralizing antibodies in cotton rats, as well as antigen-specific IFN-*γ*-producing CD4+ and CD8+ T cells in mice. These responses were comparable to or exceeding those elicited by RNA delivered by viral particles or electroporation of pDNA and provided protection against subsequent RSV infection.

## 5. Clinical Development of LNP-RNAi for Viral Infections

The exhaustive work undertaken in preclinical studies (both* in vitro* in culture cells and* in vivo* in animals) has shown that RNAi therapeutic against viral infections is highly effective at reducing virus replication and also useful as a tool to rapidly identify novel antiviral drug targets via large-scale screens for a number of viral infections [175]. Several novel viral targets have been identified and are the subject of intense research and development, but definitive evidence is lacking from well-controlled studies that demonstrate the effectiveness in these infection diseases [176]. Therefore, the next logical step must consist of human clinical trials that depict the realistic possibilities of this kind of therapy in viral infections.

The number of RNAi-based clinical trials for viral infections has grown over the past several years and has included studies against respiratory syncytial virus (RSV), HCV, HBV, HIV, and Ebola virus [177], some of them using lipid-based systems as vectors.  Table 4 collects the clinical trials evolving the application of RNAi molecules to the treatment of viral infections.

Alnylam Pharmaceuticals Inc. has carried out clinical trials with a naked siRNA against RSV nucleocapsid (ALN-RSV01). A Phase IIb study in adult lung transplant patients showed that this candidate is a promising alternative for RSV-induced bronchiolitis obliterans syndrome (BOS) that causes significant morbidity and mortality in this group of patients; moreover, the treatment with the siRNA was safe and well tolerated, and it was associated with more than 50% reduction in the incidence of new or progressive BOS at days 90 and 180 [52].

The international clinical-stage biopharmaceutical company Santaris Pharma A/S has developed an anti-miRNA drug candidate currently in clinical testing (Phase II) for treatment of HCV infections (Miravirsen, SPC3649). This drug acts against MiR-122, a liver-specific miRNA that the HCV requires for replication [53]. Data from the Phase IIa trial showed that Miravirsen was well tolerated by patients with chronic HCV genotype 1, given as weekly subcutaneous injections, over 4 weeks. Antiviral activity was continued and prolonged well beyond the end of active therapy. These data provide clinical evidence about the potential of Miravirsen as once weekly monotherapy for chronic HCV [54]. Although in a less advanced state, Benitec Biopharma Ltd., with its subsidiary Tacere Therapeutics, has designed a shRNAi-based multicassette vector called TT-034, which targets 3 well-conserved regions of HCV simultaneously, thus preventing generation of drug-resistant mutants. In addition, the targeted regions are conserved across all genotypes. Recently Benitec Biopharma Ltd. has announced that the first patient enrolled in the “first in man” Phase I/II clinical trial for TT-034 has received the first dose [55].

The biopharmaceutical company Arrowhead Research Corporation has recently presented data on the Phase I clinical study of ARC-520, the company's clinical candidate for the treatment of chronic HBV infection. ARC-520 is a siRNA-based therapeutic composed of a hepatocyte-targeted peptide (NAG-MLP) that promotes the endosomal escape of the liver tropic HBV cholesterol-siRNA. Although additional blinding results are still missing, initial results in 36 healthy volunteers receiving different doses indicate that ARC-520 is well tolerated at doses expected to be efficacious in patients with chronic HBV [178], and a Phase IIa clinical trial has recently begun [56]. The study is planned to enroll up to 16 chronic HBV patients in two-dose cohorts with patients receiving either ARC-520 or placebo in combination with entecavir. The study is designed to evaluate the depth and duration of hepatitis B surface antigen (HBsAg) decline, among other measures, in response to a single dose of ARC-520.

In the case of HIV an* ex vivo* silencing approach has been developed by the company Calimmune, by using shRNA licensed by Benitec Biopharma. T cells are extracted from HIV patients, the gene that codes for the CCR5 receptor protein is silenced *ex vivo* and re-injecting the modified cells, resistance to HIV is conferred to the patients. In its Phase l/ll clinical trial of the treatment, the enrolment of the first cohort of patients, which corresponds to the group that will receive modified CD4+ T cells and CD34+ stem cells, is about to be completed [57].

Finally, siRNA delivery against Ebola virus is also being under subject of a Phase I clinical trial recently initiated. Tekmira Pharmaceuticals has developed a therapeutic product composed of a combination of modified siRNAs targeting the Zaire Ebola polymerase, viral protein (VP) 24, and VP35 formulated in LNP (TKM-Ebola). The Ebola Phase I clinical trial is a randomized, single-blind, placebo-controlled study involving single ascending doses and multiple ascending doses of the lipid nanoparticle based formulation (TKM-Ebola). The study will assess the safety, tolerability, and pharmacokinetics of administering TKM-Ebola to healthy adult subjects [58].

## 6. Conclusions and Future Perspectives

As summarized in this review, RNAi is a promising therapeutic strategy due to its ability to silence any gene with a known sequence. However, the use of RNAi in the clinic is limited by the complexity of effective and well-controlled delivery* in vivo*. Naked RNAs have potential toxicities such as saturation of the innate RNAi machinery, stimulation of the immune response, and off-target effects. Moreover, systemic administration of RNAs encounters several obstacles that reduce their therapeutic efficacy: they are highly unstable intravascularly, with a short half-life due to their susceptibility to serum nucleases and rapid renal clearance. Consequently, RNAs do not accumulate in target tissues and cannot readily cross target cell membranes to access their cytoplasmic site of action. In addition, in the field of viral infections, it is necessary to find out new specific and effective targets and alternative combined targets to avoid mutant escapes.

The efforts to achieve a realistic clinical application of RNAi as therapeutic for viral infections should be focused mainly on three areas: reducing toxicity of RNAs while increasing stability, avoiding mutant escapes, and delivery to target tissues. RNA design and chemical modifications increase stability, reduce immunogenicity, and may help to reduce viral escapes; however, even modified, naked RNAs have poor cellular uptake due to their small size, net negative charge, renal clearance, and hydrophilicity. A range of delivery vectors such as liposomes, polymers, and nanoparticles have been developed to facilitate efficient cellular uptake and target site accumulation as well as to provide a degree of protection. Among all delivery platforms, safe and easily manufactured LNP are good candidates, which can additionally be functionalized to achieve appropriate tropism, especially when the RNAi molecules are not tissue-specific. In addition, the availability of different types of LNP favors the chance of attaining synergistic effects between RNAi molecules and conventional drugs, which may be greatly useful in the case of drugs that develop resistances or do not result effective. All these considerations, joint with data from ongoing clinical trials, suggest that in an early future the delivery of RNAi therapeutics by LNP could be a first-line treatment in several human diseases, such as viral infections.

## Figures and Tables

**Table 1:** Indications addressed by gene therapy clinical trials up to January 2014.

Ranking	Indication	Number of trials (*n*)	Percentage with respect to total gene therapy clinical trials (%)
1	Cancer diseases	1274	63.8
2	Monogenic diseases	178	8.9
3	Infectious diseases	164	8.2
4	Cardiovascular diseases	162	8.1
5	Healthy volunteers	52	2.6
6	Gene marking	50	2.5
7	Neurological diseases	37	1.9
8	Other diseases	35	1.8
9	Ocular diseases	31	1.6
10	Inflammatory diseases	13	0.7

**Table 2:** Studies carried out with lipid-based nanosystems as RNAi delivery vectors against hepatitis C (HCV).

Lipid nanosystem	RNAi	Targeting molecule	Culture cells	*In vivo* model	Reference
Cationic liposomes	siRNAs against the 5′-UTR and 3′-UTR of the HCV genome	Lactosylated-PE	FLR3-1 and R6FLR-N cells	CN2-29 transgenic mice	[33]
Cationic liposomes	HCV-core specific siRNA (siHCc)	Apo A-I	Huh7	HCV mouse model constructed by hydrodynamic injection of DNA plasmid expressing viral proteins	[34]
Cationic liposomes	HCV-core specific siRNA (siHCc)	Recombinant human apo A-I	—	HCV mouse model constructed by hydrodynamic injection of DNA plasmid expressing viral proteins	[35]
Cationic nanosomes	siRNAs against hte stem-loop domains II–IV of HCV 5′UTR	—	Huh-7.5 and R4-GFP cells	HCC tumor-xenograft mice model for HCV	[36]
Cationic LNP	sshRNA targeting the HCV IRES	—	—	Reporter mice that express in the liver firefly luciferase under the control of the HCV IRES	[37]

Lactosylated-PE: lactosylated-phosphatidylethanolamine; FLR3-1 cells: HuH-7 cells bearing an HCV subgenomic replicon (genotype 1b); R6FLR-N cells: HuH-7 cells bearing an HCV subgenomic replicon (genotype 1b); CN2-29 transgenic mice: mice that carry an HCV transgene; Apo A-I: apolipoprotein A-I; Huh7 cells: human hepatoma cell line; Huh7.5 cells: Huh7 cells that contain a mutation in RIG-I believed to be responsible for the improved replication of HCV; R4-GFP cells: IFN-*α*-resistant HCV-GFP chimer replicon cell line; HCC: hepatocellular carcinoma; LNP: lipid nanoparticles; IRES: internal ribosome entry site.

**Table 3:** Studies carried out with lipid-based nanosystems as RNAi delivery vectors against hepatitis B (HBV).

Lipid nanosystem	RNAi	Targeting molecule	Culture cells	*In vivo* model	Reference
SNALP	HBV siRNAs chemically stabilized for nuclease resistance	—	HBV-replicating HepG2	HBV mouse model constructed by hydrodynamic injection of HBV vector DNA	[47]
Cationic liposomes	HBV-X specific siRNA (siHBV)	Apo A-I	HepG2 and Huh7	Acute HBV-infected mouse model by hydrodynamic injection of a plasmid	[48]
PEGylated cationic liposomes	HBV specific siRNA	—	Huh7 cells previously transfected with HBV replication target plasmid	HBV transgenic mice	[49]
DODAG 8 lipid	HBV specific siRNA	—	—	HBV transgenic mice	[50]
Cationic liposomes	Altriol modified HBV-X siRNA	Galactose	Huh7 cells previously transfected with HBV target DNA plasmid	HBV transgenic mice	[51]

SNALP: stable nucleic acid lipid particle; HepG2: liver hepatocellular carcinoma cells; HuH7: human hepatoma cell line; DODAG: *N*′, *N*′-dioctadecyl-*N*-4,8- diaza-10-aminodecanoylglycine amide.

**Table 4:** Clinical trials evolving the application of RNAi in the treatment of viral infections.

Clinical candidate	Targeting virus	RNAi molecule	Clinical phase	Company	Reference
ALN-RSV01	RSV	siRNA	IIb	Alnylam Pharmaceuticals Inc.	[52]
Miravirsen, SPC3649	HCV	antimiRNA	IIa	Santaris Pharma A/S	[53, 54]
TT-034	HCV	shRNA	I/IIa	Benitec Biopharma Ltd.	[55]
ARC-520	HBV	siRNA	II	Arrowhead Research Corporation	[56]
CCR5 negative cells (generated *ex vivo*)	HIV	shRNA	I/II	Calimmune and Benitec Biopharma Ltd.	[57]
TKM-Ebola	Ebola virus	siRNAs	I	Tekmira Pharmaceuticals	[58]

CCR5: chemokine receptor 5; RSV: respiratory syncytial virus; HCV: hepatitis C virus; HBV: hepatitis B virus; HIV: human immunodeficiency virus; siRNA: short interfering RNA; miRNA: microRNA; shRNA: short hairpin RNA.
